# Incidental extravascular findings in CT angiograms in patients post endovascular abdominal aortic aneurysm repair: clinical relevance and frequency

**DOI:** 10.1186/s42155-018-0016-2

**Published:** 2018-07-15

**Authors:** Permesh Singh Dhillon, Mohammad Waleed Butt, Graham Pollock, James Kirk, Peter Bungay, Mario De Nunzio, Peter Thurley

**Affiliations:** 1 Clinical Radiology, Royal Derby Hospital, Derby Teaching Hospitals NHS Foundation Trust, Derby, UK; 2 Radiological Sciences, University of Nottingham, Queen’s Medical Centre, Nottingham, UK; 3 Clinical Radiology, Queens Medical Centre, Nottingham University Hospitals NHS Trust, Nottingham, NG7 2UH UK

**Keywords:** Abdominal aortic aneurysm (AAA), Endovascular aneurysm repair (EVAR), Incidental finding, CT angiogram

## Abstract

**Background:**

To evaluate the incidence and clinical relevance of extravascular incidental findings (EVIF), particularly malignancies, in planning and follow-up CT angiograms (CTA) of the abdominal aorta in patients who underwent endovascular aneurysm repair (EVAR) of abdominal aortic aneurysm. Retrospective study of 2203 planning and follow-up CTAs of 418 patients who underwent EVAR in a single tertiary centre between 2006 and 2015. CTA reports were scrutinized for EVIFs, which were classified according to clinical relevance, into (I) immediate, (II) potential and (III) no clinical relevance. Clinical follow-up and management were reviewed for significant findings. Follow-up CTAs of patients with incidental malignancies were re-reviewed by two consultant radiologists to evaluate if early missed malignant findings on previous CTAs were present.

**Results:**

In total, 950 EVIFs were noted in 418 patients [31 females (7.4%), 387 males (92.6%); age range 63–93, mean age 79.0 years]. The number of patients with findings in each category were; Category I (115), Category II (165), Category III (304). Incidental malignant findings were reported in 51 patients (12.2%), of which 27 were noted on the initial CTA (6.5%) and 24 on follow-up CTAs (5.7%). Of the 24 patients with malignancies on follow-up CTAs, 13 had early malignant findings missed or misinterpreted on previous CTAs, while 11 had no significant abnormality even on retrospective review.

**Conclusion:**

A high number of significant EVIFs, particularly incidental malignancies, can be identified in follow-up CTAs of patients who undergo EVAR. Specific ‘review areas’ when reporting surveillance CTAs can be recommended based on the findings of our study.

## Background

Endovascular aneurysm repair (EVAR) has become the predominant treatment option for patients with abdominal aortic aneurysm (AAA) accounting for 78% of all elective repair in the United States in 2010 (Dua et al. 2014). In addition, recent large multi-centre randomised trials have demonstrated similar short-term outcomes between EVAR and open surgical repair for the treatment of ruptured AAA which may further increase endovascular management (Trial et al. 2009; Hoornweg et al. 2007).

Despite superior perioperative mortality outcomes and similar long-term survival compared to open surgical repair (Lederle et al. 2009; Greenhalgh et al. 2004), EVAR is beset by its higher rates of observed complications and need for secondary intervention (De Bruin et al. 2010; Hobo et al. 2006). Indications for re-intervention often include stent migration and endoleak with the associated risk of subsequent aneurysm rupture. Life-long surveillance is therefore recommended by multiple societies (Walker et al. 2010). Computed tomography angiography (CTA) remains the most widely used imaging modality for the purposes of pre-operative planning and surveillance due to its availability, high-throughput, reproducibility, contrast resolution and volumetric multi-planar reconstruction functionality. Although there is no consensus on surveillance frequency and modality, commonly used protocols traditionally incorporated arterial phase CTA imaging at 1, 6 and 12 month periods post-procedure and subsequent yearly follow-up (Hirsch et al. 2006). Several studies have demonstrated this can be done with similar sensitivity and specificity for endoleak detection as multiphase CT (Iezzi et al. 2006; Macari et al. 2006). Furthermore, some authors advocate the sole use of annual Doppler ultrasound for endoleak detection if no complications have been demonstrated on CTA at 12 months post-procedure (Chaer et al. 2009; Sternbergh 3rd et al. 2008).

Incidental findings are commonplace in clinical radiology and whilst they may lead to early significant diagnoses, over-diagnosis with unnecessary procedural or imaging work up and psychological distress to patients can also occur. Extravascular structures are readily depicted on CTA and previous studies demonstrated the prevalence of clinically significant incidental findings to be in the range of 5.6 and 12% (Katz et al. 1999; McDougal et al. 2006). Interestingly, higher rates of detection were shown in more recent studies which focussed on lower limb run off CTA with figures as high as 27% in patients presenting with acute limb ischaemia (Preuss et al. 2015). Therefore, our objectives were to primarily evaluate the frequency and clinical relevance of extravascular incidental findings (EVIF) in the post EVAR patient sub-set who undergo CTA and secondarily to assess reporting accuracy by retrospectively scrutinising prior images for evidence of early disease in cases where malignant findings had been demonstrated.

## Methods

### Patient population

A retrospective review of 2203 planning and surveillance CTAs of 418 patients who underwent EVAR (elective and emergency) in a single tertiary centre between 2006 and 2015 were included in this study. No IRB approval was required. No patients were excluded. Basic demographic data of age and gender was obtained. Each patient had at least a planning and surveillance CTA performed. Surveillance CTAs were obtained at 1, 6, and 12 monthly intervals unless follow up was converted to ultrasound imaging or if the patient had deceased.

### Image acquisition

Images were obtained on a 64 multi-detector CT system (Toshiba Aquilion). The patient was positioned supine with arms above their head and the scan range was from above the diaphragms (lung bases) to the lesser trochanters. Images were acquired in the arterial phase after injection of 100 ml of intravenous contrast material (Omnipaque® 300, GE healthcare). The following acquisition parameters were used: 120 kV, reference tube 182mAs, 0.5 s rotation time, helical pitch 53. The slice thickness was 1 mm and sections were reconstructed in the axial, coronal and sagittal planes of 3 × 3 mm.

### EVIF definition

EVIF was defined as any finding that was previously unknown to the reporting radiologist, which was not included in the scan request forms or previous CTAs. This included, for example, a known lymphoma to the clinician but not the radiologist. Scan request details were reviewed to identify any known conditions and final reports were scrutinized using the patient archiving and communication system (PACS), and any EVIF noted was included in the data collection. Only the first reported EVIF was included and any similar EVIFs in subsequent scans were excluded.

### EVIF classification

EVIFs were classified according to a previous study and the White Paper of the American College of Radiology (ACR) Incidental Findings Committee 2010 based on clinical relevance, into (I) immediate, (II) potential and (III) no clinical relevance (Preuss et al. 2015; Berland et al. 2010). Immediate clinical relevance (Class I) was defined as any finding that required urgent intervention, treatment or follow-up and included any highly suspicious malignant findings and infective sources that may lead to severe morbidity or mortality. Class II or potential clinically relevant findings were identified if the finding may lead to future morbidity and could require follow up, for example, including small pulmonary nodules (<1 cm) and non-obstructing renal stones. Findings with no clinical relevance (Class III) did not require follow up or change in management, such as simple renal and hepatic cysts or degenerative lumbar spine.

### Data analysis

All scans were reported by any one of six consultant vascular radiologists and retrospective review of malignant findings on surveillance CTA was undertaken by two of the same six consultant vascular radiologists with 7 and 20 years of consultant experience. Incidental malignant findings were classified into two groups; planning (if identified on the first scan) or surveillance CTA (identified on any follow up scans) and then further categorized into missed or non-visible findings following retrospective review. Clinical follow up, diagnoses and management were reviewed for significant findings and were based on electronic records of clinic letters, further imaging, histopathology and biochemistry results and drug charts. All data was computed and analysed using Microsoft Excel 2011 V14.2.

## Results

In our study, a total of 950 EVIFs were noted in 2203 CT scans of 418 patients (31 females (7.4%), 387 males (92.6%); age range 63–93, mean age 79.0 ± 6.6(SD) years; mean scans per patient 5.3 ± 2.8(SD)). There were 115 patients (27.5%) with 144 Class I findings, 165 patients (39.5%) with 209 Class II findings and 304 patients (72.7%) with 597 Class III findings. At least one finding was noted in 362 patients (86.6%) and 38 patients (9.1%) had findings in all three categories.

All findings were classified according to three anatomical areas; Chest, Abdomen, Musculoskeletal (MSK). In Class I, there were 100 (69.2%) significant EVIFs in the abdomen, 42 (29.4%) in the chest and 2 (1.4%) were MSK. Of 144 Class 1 findings, follow up information was available for 115 EVIFs (79.9%) while 29 were unknown. These findings are summarised in Table 1.

**Table 1:** Class I extravascular incidental findings according to system area. (MSK denotes musculoskeletal)

System	Incidental findings	Number (relative Frequency %)	Follow-up
Chest	Lung mass/cancer	9 (6.3)	8 cancers confirmed, 1 benign
	Pericardial effusion	2 (1.4)	1 treated, 1 died
	Pleural effusion (moderate/large)	5 (3.5)	3 treated, 2 unknown
	Pneumonia	24 (16.8)	6 treated, 3 asymptomatic, 15 unknown
	Pulmonary embolus	2 (1.4)	2 treated
Abdomen	Abdominal ascites	1 (0.7)	Cardiac failure
	Acute pancreatitis	2 (1.4)	Treated
	Adrenal lesion (suspicious)	1 (0.7)	Stable
	Appendicitis	1 (0.7)	Unknown
	Bladder wall thickening/cancer	17 (11.9)	9 cancers confirmed, 8 benign, 1 unknown
	Bone metastasis	3 (2.1)	3 confirmed
	Carcinoid tumour	1 (0.7)	Confirmed
	Colorectal mass/cancer	24 (16.8)	10 cancers confirmed, 14 benign
	Cholecystitis	4 (2.8)	3 treated, 1 unknown
	Colovesical fistula	1 (0.7)	Treated
	Diverticulitis	2 (1.4)	Unknown
	Gallbladder mass/cancer	1 (0.7)	Cancer confirmed
	Gastric wall thickening/cancer	4 (2.8)	2 cancers confirmed, 2 benign
	Hydronephrosis (moderate/severe)	11 (7.4)	3 stented, 1 chronic, 7 unknown
	Liver mass/cancer	4 (2.8)	3 metastasis, 1 benign
	Pancreatic mass/cancer	2 (1.4)	2 cancers confirmed
	Prostate mass/cancer	4 (2.8)	4 cancers confirmed
	Pyelonephritis	1 (0.7)	Treated
	Renal mass/cancer	8 (5.6)	3 cancers confirmed, 5 benign
	Splenomegaly	1 (0.7)	Known Non-Hodgkin’s Lymphoma
	Splenic lesion (suspicious)	1 (0.7)	Likely haemangioma, surveillance
	Strangulated inguinal hernia	1 (0.7)	Unknown
	Widespread lymphadenopathy	5 (3.5)	4 Non-Hodgkin’s lymphoma
MSK	Osteomyelitis	1 (0.7)	Unknown
	Pathological humeral fracture	1 (0.7)	Bone metastasis
Total		144	

Incidental malignant findings, which accounted for the largest combined group of EVIFs in Class I, were reported in 51 patients (12.2%) [49 males, 2 females, mean age 80.9 years], of which 27 were noted on the planning CTA and 24 on follow-up CTAs. Of the 24 patients, 13 had early malignant findings missed or misinterpreted on previous CTAs, while 11 had no significant abnormality even on retrospective review (Table 2). Retrospectively, 2 of the 13 missed malignant findings were originally identified but dismissed as benign, which included a tiny lung base lesion later diagnosed as bronchogenic lung carcinoma and pancreatic duct dilatation initially felt to be due to chronic pancreatitis, which ultimately proved to be secondary to pancreatic malignancy. There was a 100% inter-rater agreement between both radiologists in assessing the malignant findings retrospectively in surveillance CTAs.

**Table 2:** Extravascular incidental malignant findings on planning and surveillance CT Angiogram

Type of cancer	Number (Relative frequency %)			
	Planning CTA	Surveillance CTA		Total
		Non-visible	Missed	
Bladder	6 (22.2)	0	3 (23)	9 (17.6)
Bone	1 (3.7)	1 (9.1)	1 (7.7)	3 (5.9)
Carcinoid	1 (3.7)	0	0	1 (1.9)
Colorectal	7 (25.9)	2 (18.2)	1 (7.7)	10 (19.6)
Gallbladder	0	1 (9.1)	0	1 (1.9)
Gastric	1 (3.7)	1 (9.1)	0	2 (3.9)
Liver	0	0	3 (23)	3 (5.9)
Lung	4 (14.8)	3 (27.2)	1 (7.7)	8 (15.7)
Non Hodgkin’s Lymphoma	3 (11.1)	1 (9.1)	1 (7.7)	5 (9.8)
Pancreatic	0	1 (9.1)	1 (7.7)	2 (3.9)
Prostate	2 (7.4)	1 (9.1)	1 (7.7)	4 (7.8)
Renal	2 (7.4)	0	1 (7.7)	3 (5.9)
Total	21	11	13	51

**Fig. 1:**
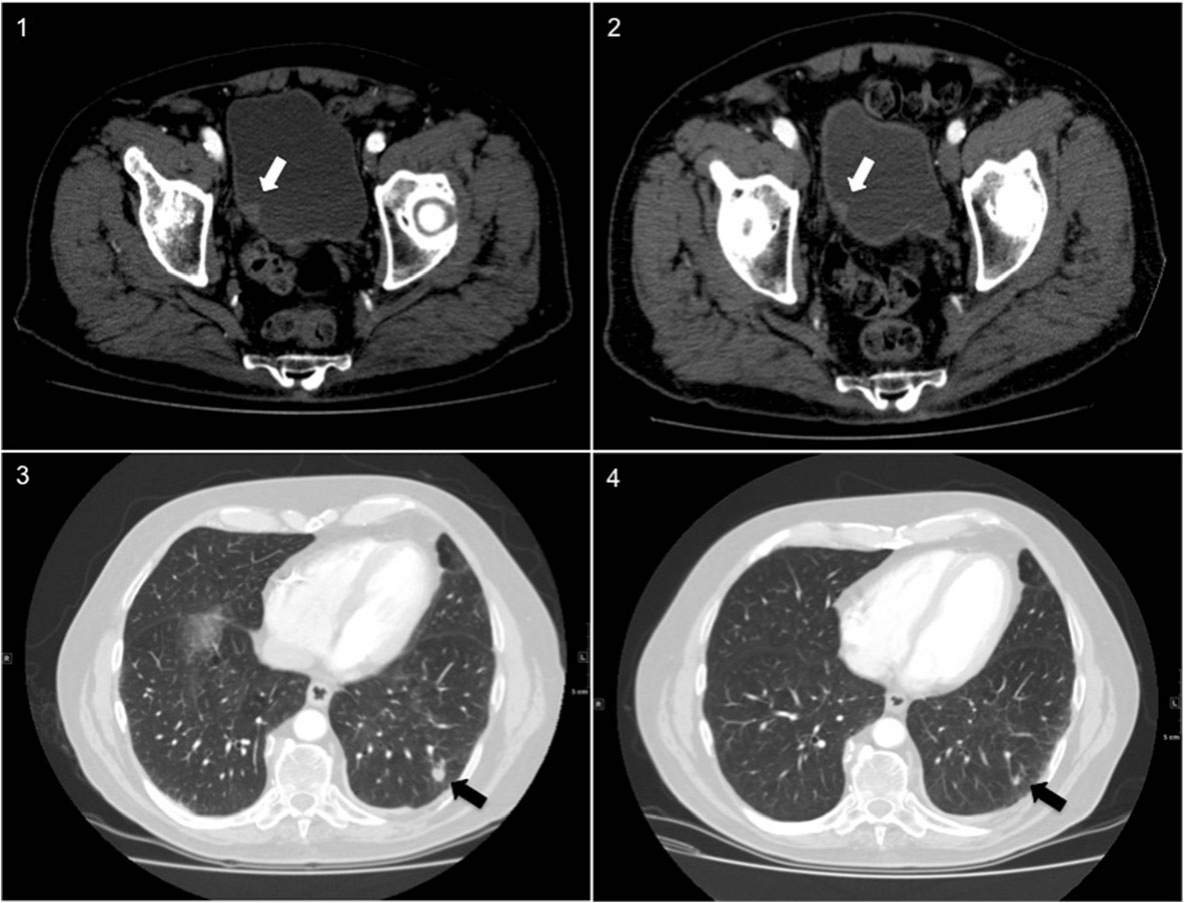
Example images of missed malignant findings on surveillance CT Angiogram post endovascular aortic aneurysm repair. Axial slice of CTA showing the urinary bladder right lateral wall thickening (white arrow), confirmed bladder transitional cell carcinoma at diagnosis (**1**) and retrospectively on a previous scan (**2**). Axial slice of CTA showing the 11 mm left lung lower lobe lesion (black arrow), confirmed adeno-squamous lung cancer at diagnosis (**3**) and retrospectively on a previous scan (**4**), which was initially considered benign

There was a spread of 12 different malignancies identified. The highest overall frequencies of incidental malignancies were colorectal cancer (10), urinary bladder cancer (9) and lung cancer (8). The distribution of colorectal cancers included four caecal and two each in the ascending colon, sigmoid and rectum. Amongst the 27 malignant findings identified on the planning CTA, the most frequent were colorectal (7) and urinary bladder (6) cancers. Urinary bladder cancer (3) and liver metastasis (3) were the most common missed malignant findings in retrospect. The others included pancreatic cancer (1), renal cancer (1), lung cancer (1), colorectal cancer (1), prostate cancer (1), bone metastasis (1) and Non-Hodgkin’s Lymphoma (1). Three lung and two colorectal cancer cases were the most frequent malignancies that could not be identified on previous scans even in retrospect. Figure 1 includes examples of subtle missed early malignant findings.

The most common Class II EVIFs included gallstones (102), small pleural effusions (24) and renal calculi (20). Class III EVIFs were most frequently identified as uncomplicated diverticulosis (147), simple renal cysts (101) and degenerative lumbar spine (68). The remaining EVIFs are summarised in Tables 3 and 4 respectively.

**Table 3:** Class II extravascular incidental findings according to system area. (MSK denotes musculoskeletal)

System	Incidental findings	Number (relative frequency %)	Follow-up
Chest	Cardiomegaly	3 (1.4)	
	Coronary artery calcification	1 (0.5)	
	Pleural effusion (small)	24 (11.4)	
	Pleural nodule (<1 cm)	2 (1.0)	2 benign
	Pulmonary nodules (<1 cm)	3 (1.4)	3 no growth
	Rib fracture	1 (0.5)	No intervention
Abdomen	Appendix mucocoele	1 (0.5)	
	Bladder calculi	3 (1.4)	
	Chronic pancreatitis	1 (0.5)	
	Dilated common bile duct	8 (3.8)	6 no cause, 2 gallstones
	Gallstones	102 (48.5)	
	Hydrocoele	1 (0.5)	No change
	Hydronephrosis (mild)	3 (1.4)	
	Incarcerated hiatus hernia	2 (1.0)	2 no intervention
	Lymphadenopathy (coeliac/mediastinal)	5 (2.3)	
	Meningocoele (S2 neural foramina)	1 (0.5)	No intervention
	Ovarian dermoid	1 (0.5)	
	Pancreatic duct dilatation	1 (0.5)	No intervention
	Pancreatic pseudocyst	8 (3.8)	8 no intervention
	Prostate hypertrophy	10 (4.7)	
	Renal stone	20 (9.5)	
	Uterine fibroids	1 (0.5)	
MSK	Hip osteoarthritis	2 (1.0)	
	L5/S1 spondylolisthesis	3 (1.4)	
	Paget’s of hemipelvis	1 (0.5)	
	Sacral sclerotic change	1 (0.5)	No intervention
Total		209

**Table 4:** Class III extravascular incidental findings according to system area. (MSK denotes musculoskeletal)

System	Incidental findings	Number (relative frequency %)
Chest	Bronchiectasis	16 (2.7)
	Diaphragmatic hernia	3 (0.5)
	Emphysema	58 (9.7)
	Lung fibrosis	31 (5.2)
	Pleural plaque	25 (4.2)
	Pulmonary atelectasis	14 (2.3)
Abdomen	Abdominal wall hernia	4 (0.6)
	Adrenal adenoma	10 (1.6)
	Diverticular disease	147 (24.6)
	Epigastric hernia	1 (0.2)
	Hiatus hernia	18 (3)
	Horseshoe kidney	1 (0.2)
	Incisional hernia	1 (0.2)
	Inguinal hernia	21 (3.5)
	Inguinal lymphocoele	1 (0.2)
	Liver haemangioma	1 (0.2)
	Lymphocoele	1 (0.2)
	Mesenteric panniculitis	2 (0.3)
	Ovarian cyst	1 (0.2)
	Pancreatic calcification	2 (0.3)
	Parastomal hernia	1 (0.2)
	Simple liver cyst	56 (9.4)
	Simple renal cyst	101 (16.9)
	Spigelian hernia	1 (0.2)
	Splenunculi	1 (0.2)
MSK	Degenerative lumbar spine	68 (11.4)
	Lumbar wedge fracture	4 (0.6)
	Thoracic wedge fracture	6 (1.0)
	Vertebral body haemangioma	1 (0.2)
Total		597

## Discussion

A high number of significant EVIFs can be identified in follow-up CTAs of patients who undergo EVAR, which is of importance in this higher risk cohort of elderly patients with multiple co-morbidities. Many of these EVIFs were followed up and were shown to cause a change in the management of patients by aiding early diagnoses.

This study included a large sample of patients (418) and scans (2203) compared to previous studies, ranging from 82 to 290 patients (Preuss et al. 2015; Tornqvist et al. 2016; Naidu et al. 2010; Indes et al. 2008; Waqas et al. 2014; Gufler et al. 2014). The incidence of EVIFs in Class I was higher than most previous studies (range 6.5% – 23.7%), but lower than 37 and 42% in Tornqvist et al. and Indes et al.’s studies respectively (Katz et al. 1999; McDougal et al. 2006; Preuss et al. 2015; Tornqvist et al. 2016; Naidu et al. 2010; Indes et al. 2008; Waqas et al. 2014; Gufler et al. 2014). In our study, classification of EVIFs was similar to Preuss et al.’s study, which explains the comparable 27% of EVIF incidence (Preuss et al. 2015). However, the lack of standardisation of EVIF definitions and differences in EVIF classification across other studies make the results less directly comparable. Difference in the scanner type (single helical CT) and inclusion of the venous phase in Katz et al.’s and Naidu et al.’s studies respectively, may have also influenced the findings (Katz et al. 1999; Naidu et al. 2010).

The marked gender imbalance (92.6% males) in the study could possibly explain the few gynaecological pathologies detected while the lack of EVIFs in the MSK region compared with other studies could be accounted for by the total area imaged that included lower limbs in other publications (Preuss et al. 2015; Naidu et al. 2010). Overall findings of high frequencies of pneumonia, gallstones, simple renal and hepatic cysts, diverticular disease and degenerative lumbar spine in each class were in line with previously reported studies (Preuss et al. 2015; Waqas et al. 2014; Iezzi et al. 2007). However, our follow-up rate (80%) for important EVIFs was significantly better than other studies (40% reported in Preuss et al., 58% in Naidu et al. and 73% in McDougal et al.) (McDougal et al. 2006; Preuss et al. 2015; Naidu et al. 2010).

Most lung consolidative changes were identified on the first post-EVAR CTA and were likely due to secondary complications of hospital admissions. Patients who were asymptomatic at the clinical follow up required no intervention. A large number of patients who underwent further work up for suspicious lesions yielded malignant results (51 of 85). This highlights the importance of a low threshold for further investigation of patients who had Class I EVIFs diagnosed on surveillance CTAs.

In our study, there was a higher incidence of incidental malignancies (12.2%) compared to previous studies (range 0–5.2%) (Preuss et al. 2015; Tornqvist et al. 2016; Naidu et al. 2010; Gufler et al. 2014; Iezzi et al. 2007; Belgrano et al. 2010; Prabhakar et al. 2015; Hughes et al. 2016; Ho et al. 2016). This could be attributed to the nature of our study, which included surveillance CTAs over an extended period and larger number of scans. It can be postulated that the greater malignant findings detected was more likely in an elderly age group with higher co-morbidities, although similar patient demographics were observed in Preuss et al., Tornqvist et al., Gufler et al., and Indes et al.’s studies (mean age 74.9, 78.7, 81.6, and 76 respectively) (Preuss et al. 2015; Tornqvist et al. 2016; Indes et al. 2008; Gufler et al. 2014). Certain malignancies were also known to clinicians at diagnosis (10 of 51) but not included in the request reports and were unknown to the reporting radiologists. However, this was still relevant and included as it remained a new diagnosis to the radiologist.

The high frequency of urinary bladder cancer in the cohort was an intriguing observation while the high number of colorectal cancers was unsurprising.

A possible explanation for the commonly missed liver malignancy could be the limitation of an arterial phase scan in detecting and characterising liver lesions. Some missed early malignant findings were also very subtle in retrospect and could be easily overlooked or dismissed as a benign finding at the time of reporting. However, it is difficult to draw conclusions due to the small number of cases. All retrospectively missed incidental malignant findings were discussed at the departmental discrepancy meeting and any unexpected findings were escalated in accordance with the Royal College of Radiologists (RCR) and General Medical Council (GMC) Duty of Candour statements (The professional duty of candour 2015).

A limitation of our study included a possible underestimation of EVIFs as only the final scan reports were reviewed. However, as the surveillance scans were reported by experienced consultant radiologists and any previous scans were compared during reporting, the number of undetected EVIFs should be limited in number. Follow up data did not include paper medical records, which accounted for the incomplete clinical follow up rates. A few patients had also died prior to a full work up or follow up. While an interesting aspect of our study included the retrospective review of incidental malignancies on surveillance CTA, our results may have been biased by the reviewing radiologists having prior knowledge of the clinical diagnosis.

## Conclusion

A significant amount of EVIFs, particularly incidental malignancies, can be detected in surveillance CTAs of patients post-EVAR. Hence, it is prudent to be vigilant in evaluation of abdominal CTAs and necessary clinical follow-up arranged. Comprehensive overview of checklist areas with particular attention to the liver and bladder, which were commonly missed sites for early pathology, can be suggested on the basis of our findings. It remains to be seen if change in patients’ management following important EVIF detection and arguably earlier diagnoses, significantly translates into improved patient outcome. Future work may include the cost analysis and radiation exposure of follow up imaging and potential procedures.

## Abbreviations

AAA: Abdominal Aortic Aneurysm
CT: Computed Tomography
CTA: Computed Tomography Angiography
EVAR: Endovascular Aortic Repair
EVIF: Extravascular Incidental Finding
GMC: General Medical Council
MSK: Musculoskeletal
RCR: Royal College of Radiologists
